# KLF4 Affects Acute Renal Allograft Injury via Binding to MicroRNA-155-5p Promoter to Regulate ERRFI1

**DOI:** 10.1155/2022/5845627

**Published:** 2022-03-17

**Authors:** Jiquan Zhao, Jiqiang Zhao, Zhaohui He, Minzhuan Lin, Feng Huo

**Affiliations:** ^1^Department of Urological Surgery, The Eighth Affiliated Hospital of Sun Yat-sen University (Shenzhen Futian), Shenzhen, 518033 Guangdong, China; ^2^Department of Organ Transplantation, The Third Affiliated Hospital of Guangzhou Medical University, 3 Duobao Road, Liwan District, Guangzhou, 510150 Guangdong, China; ^3^Department of Hepatobiliary Surgery, General Hospital of the PLA Southern Theater Command, Guangzhou, 510010 Guangdong, China

## Abstract

Kruppel-like factor 4 (KLF4) owns the promising potential in treating kidney injury, which inevitably occurs during renal allograft. Given that, this research targets to unveil KLF4-oriented mechanism from microRNA-155-5p/ERBB receptor feedback inhibitor 1 (miR-155-5p/ERRFI1) axis in acute renal allograft injury. Mice were injected with miR-155-5p-related sequences before acute renal allograft modeling. Afterwards, serum inflammation, along with oxidative stress, renal tubular injury, and apoptosis in renal tissues were detected. HK-2 cells were processed by hypoxia/reoxygenation (H/R) and transfected with miR-155-5p- or ERRFI1-related sequences, after which cell proliferation and apoptosis were measured. KLF4, miR-155-5p, and ERRFI1 expressions and their interaction were tested. KLF4 and miR-155-5p levels were enhanced, and ERRFI1 level was repressed in mice after acute renal allograft and in H/R-treated HK-2 cells. KLF4 bound to the promoter of miR-155-5p. Depleting miR-155-5p reduced serum inflammation and attenuated oxidative stress, renal tubular injury, and apoptosis in mice with acute renal allograft injury. Downregulating miR-155-5p facilitated proliferation and repressed apoptosis of H/R-treated HK-2 cells. miR-155-5p targeted ERRFI1. Knocking down ERRFI1 antagonized the effects of downregulated miR-155-5p on acute renal allograft injury, as well as on H/R-treated HK-2 cell proliferation and apoptosis. A summary displays that silencing KLF4 suppresses miR-155-5p to attenuate acute renal allograft injury by upregulating ERRFI1, which provides a way to control acute renal allograft injury.

## 1. Introduction

Renal transplantation is the best treatment to improve the survival rate of patients with end-stage kidney disease [1]. Unfortunately, acute kidney injury (AKI), along with subsequent chronic allograft dysfunction, occurs unexpectedly during renal allograft, which may eventually progress to renal function loss [2]. Ischemic AKI is the most common complication following renal transplantation, which may be resulted from ischemia/reperfusion injury (I/RI) [3]. Moreover, current therapy is impossible to immediately predict acute renal allograft injury after renal transplantation [4]. Hence, the potential targets to manage acute renal allograft injury are urgently asked.

Kruppel-like factor 4 (KLF4) has been suggested as a therapeutic target in the context of chronic kidney disease [5]. Investigated by a late research, an increase presents in KLF4 expression in the kidneys of mice after I/RI [6]. Functionally, KLF4 downregulation has been proved to attenuate renal function deterioration after I/RI [7]. There is a self-activating and feedback mechanisms between transcription factors (including KLF4) and miRNAs [8], and KLF4 positively modulates miR-200b and miR-183 levels [9]. As the reported researches proved, regulation of miRNA is conducive to attenuate I/RI during renal allograft, such as miR-378 [10]. As to miR-155, it is implied that overexpressed miR-155 is substantially connected with abnormality in human solid organ allografts and rat renal allografts [11]. Actually, enhanced miR-155 level in plasma of renal allograft recipients is linked to renal dysfunction [12]. Moreover, abnormally expressed miR-155 is promoted to stimulate inflammation and apoptosis of tubular epithelial cells in AKI [13]. In fact, the abundance of miR-155 level in hypoxia-reoxygeneration- (H/R-) treated cells disrupts the balance of renal cell apoptosis and proliferation [14]. KLF4 as a transcription factor has not yet been studied as to regulation of miR-155-5p.

ERBB receptor feedback inhibitor 1 (ERRFI1) has been studied as a target of miRNAs to modulate AKI, and inhibited ERRFI1 deteriorates the development of AKI through interfering renal cell apoptosis and inflammation [15]. Moreover, ERRFI1 has the ability to mediate IR/I-induced oxidative stress and apoptosis [16]. To our best knowledge, a little few study has revealed the combined function of miR-155-5p and ERRFI1 in diseases.

Consulted from the aforementioned researches, we know that KLF4 is of therapeutic significance in treating kidney injury. Given that, we would like to figure out whether mediating KLF4 could manage acute renal allograft injury from the axis of miR-155-5p/ERRFI1 and hope to provide a theoretical reference for treat kidney injury.

## 2. Methods and Materials

### 2.1. Ethics Statement

This study was approved by the ethics committee of the Eighth Affiliated Hospital of Sun Yat-sen University (Shenzhen Futian). Animal treatments were reviewed and supervised by the Animal Ethics of the Eighth Affiliated Hospital of Sun Yat-sen University (Shenzhen Futian). The sufferings for animals were minimized.

### 2.2. Establishment of Acute Renal Allograft Model and Animal Treatment

Female C57BL/6 mice (14 weeks old, 24-28 g) were accessible from SLAC Laboratory Animal (Shanghai, China). There were 25 donor mice and 25 receptor mice. A model of renal allograft was established as previously described [17]. In short, a midline incision was performed on mice who had been anesthetized by 2% isoflurane (RWD Life Science Co., Ltd., Shenzhen, China). The left kidney, aorta, inferior vena cava, and ureter of donor mice were cut off under a microscope. The donor kidney was transplanted below the primary renal artery in the recipient mice after left nephrectomy, the inferior renal artery, and inferior vena cava were anastomosed perfectly with the recipient mice. Then, the ureter was anastomosed with the bladder to reconstruct urethra. After renal allograft, mice were treated with long-term ischemia (cold ischemia for 60 min and warm ischemia for 60 min) to induce ischemic injury of allograft. The mice for sham operation were treated with left kidney reanastomosis after left nephrectomy. Antagomir-negative control (NC), miR-155-5p antagomir, and si-ERRFI1 (2.5 mg/kg) were intravenously injected into mice before renal allograft (*n* = 5/group). On the next day, a model of acute renal allograft was established [18].

### 2.3. Enzyme-Linked Immunosorbent Assay (ELISA)

After 10 d [19], mice were anesthetized to obtain serums, in which proinflammatory cytokine interferon- (IFN-) *γ*, tumor necrosis factor- (TNF-) *α*, and interleukin- (IL-) 2 contents were evaluated by an ELISA kit (R&D Systems, Minneapolis, MN, USA) as the biomarkers of allograft function.

### 2.4. Renal Function Assessment

Serum creatinine content was measured by sarcosine oxidase enzymatic assay (Kehua Dongling Diagnostic Products, Shanghai, China) while estimated glomerular filtration rate (eGFR) by abbreviated Modification of Diet in Renal Disease equation. The blood samples were subjected to centrifugation at 3600 rpm to take the supernatant, which was reacted with a mixture of creatine hydrolase, sarcosine oxidase, and catalase. The optical density value (OD_546 nm_) was measured.

### 2.5. Hematoxylin-Eosin (H&E) Staining

Mice were euthanized to harvest their kidneys. Specially, the mice were anesthetized by 2% isoflurane and then were decapitated and killed. One-half of the renal allografts was immediately fixed in formalin buffer and embedded in paraffin, while the other half was frozen in liquid nitrogen. H&E staining was utilized to evaluate the renal tissue sections.

### 2.6. Transferase-Mediated Deoxyuridine Triphosphate-Biotin Nick End Labeling (TUNEL) Staining

Cell apoptosis was assessed by TUNEL staining (Roche, Mannheim, Germany). TUNEL-positive cells were counted in five random fields under a fluorescence microscope. Apoptosis index was measured as the percentage of TUNEL-positive cells in total nucleus.

### 2.7. Oxidative Stress-Related Parameter Detection

Oxidative stress in the kidneys was evaluated by measuring malondialdehyde (MDA) content and superoxide dismutase (SOD) activity. MDA content was measured with a kit (Beyotime, Shanghai, China) via thiobarbituric acid method and the OD_535 nm_ value was determined. The SOD assay kit (Beyotime) was applied to test SOD activity in the kidney [20].

### 2.8. Cell Culture and Modeling

Immortalized human renal proximal tubule (HK-2) cells, with phenotypic and functional characteristics of proximal tubule cells, were obtained from the American Type Culture Collection (VA, USA). HK-2 cells were hatched in a culture system of Dulbecco's Modified Eagle Medium/F12 supplemented with 10% fetal bovine serum (Invitrogen, Carlsbad, USA), 100 U/mL penicillin G, 100 *μ*g/mL streptomycin and 0.25 *μ*g/mL amphotericin B (Invitrogen). Then, the cells were cultivated in a hypoxic environment (1% O_2_, 94% N_2_, and 5% CO_2_) for 24 h and then in an aerobic environment (21% O_2_, 74% N_2_, and 5% CO_2_) for 3 h to establish an *in vitro* H/R model [21].

### 2.9. Cell Transfection

Overexpression- (Oe-) KLF4, oe-negative control (NC), sh-KLF4, sh-NC, inhibitor-NC, miR-155-5p inhibitor, and si-ERRFI1 were synthesized by RiboBio (Guangzhou, China) and transfected into cells via Lipofectamine 2000 (Invitrogen). The transfection efficiency was verified 48 h after cell transfection. The cells that had been transfected for 48 h were collected for *in vitro* experiments.

### 2.10. Cell Counting Kit- (CCK-) 8 Assay

CCK-8 assay was applied to assess the proliferation of HK-2 cells. HK-2 cells (10 *μ*L/well) were added with CCK-8 solution at the 2^nd^, 6^th^, and 12^th^ hour, respectively, and incubated for another 2 h. The OD_450 nm_ value was recorded by a microplate reader (Bio-Rad, Hercules, USA).

### 2.11. Flow Cytometry

HK-2 cells resuspended in phosphate-buffered saline were successively hatched with Annexin V-fluorescein isothiocyanate (5 *μ*L) and with propidium iodide (10 *μ*L) (both from Beyotime) without light exposure. Cell apoptosis was examined by a flow cytometer (FACSCalibur; BD Biosciences, Franklin Lakes, USA).

### 2.12. Reverse Transcription Quantitative Polymerase Chain Reaction (RT-qPCR)

Total RNA was extracted from tissues and cells by TRIzol reagent (Invitrogen). Total RNA (1 *μ*g) was subjected to reverse transcription by Superscript II reverse transcriptase (Invitrogen) and random primer oligonucleotides (Invitrogen). The gene-specific TaqMan miRNA detection probe (Applied Biosystems, Foster City, USA) was applied to analyze miRNA expression. Total RNA (1 mg) was reverse-transcribed through avian myeloblastosis virus (Takara, Kyoto, Japan) and stemi-loop RT primers (Applied Biosystems). The primers are shown in Table 1. Real-time PCR was conducted via 7900 HT Real-time PCR system, and gene expression levels were calculated with 2^-*ΔΔ*Ct^ method. Briefly, after an initial denaturation step at 95°C for 3 min, the amplifications were carried out with 40 cycles at a melting temperature of 95°C for 15 s and an annealing temperature of 62°C for 34 s. KLF4 and ERRFI1 expressions were normalized to glyceraldehyde-3-phosphate dehydrogenase (GAPDH) while miR-155-5p expression to U6.

### 2.13. Western Blot Assay

Total protein of tissues and cells was extracted, of which the concentration was measured by bicinchoninic acid kit (Boster, Wuhan, China). Boiled (30 *μ*g/well) with the loading buffer at 95°C, protein samples were isolated by 10% polyacrylamide gel (Boster), electroblotted onto polyvinylidene fluoride membrane, and sealed in 5% bovine serum albumin. Afterwards, the membrane was probed with primary antibodies Bax, Bcl-2, ERRFI1 (1 : 1000, Abcam, Cambridge, UK) and GAPDH (1 : 2000, Jackson Immuno Research, Pennsylvania, USA), and peroxidase-labeled secondary antibody (1 : 500, Jackson Immuno Research). Processed by the Odyssey dual-color infrared fluorescence scanning imaging system, the protein bands were assessed by Quantity One image analysis software to measure the gray value. The ratio of gray value in each target band to that in internal control band was measured.

### 2.14. Dual Luciferase Reporter Gene Assay

ERRFI1 luciferase vector was cloned into psiTM-Check2-control vector (GenePharma, Shanghai, China). The wild-type (WT) psitm-check2-ERRFI1-3′-untranslated region containing the predicted miR-155-5p site was generated. A mutant (MUT) miR-155-5p binding site plasmid was also cloned. All cloned plasmids were identified by sequencing (TsingKe, China). HK-2 cells were cotransfected with ERRFI1 WT or MUT and miR-155-5p mimic or mimic-NC via Lipofectamine 2000 (Thermo Fisher Scientific, MA, USA). The miR-155-5p promoter containing KLF4 binding sites was cloned into pGL3-Basic reporter vectors (Promega, WI, USA). HK-2 cells were cotransfected with luciferase vectors and high/low KLF4. A dual luciferase reporter system (Promega) was employed to measure luciferase activities.

### 2.15. Chromatin Immunoprecipitation (ChIP) Assay

The binding affinity of KLF4 and miR-155-5p was determined by ChIP assay based on the protocol (Beyotime). KLF4 level was increased or decreased by oe-KLF4 or sh-KLF4 vectors, respectively. HK-2 cells (1 × 10^6^ cells) were processed by ultrasound for 48 cycles, after which cell supernatant was extracted by centrifugation. Followed by that, the beads were reacted with the target protein antibody or immunoglobulin G (IgG). The antibody-bound beads were incubated with the sample to bind the antibody to the target protein. KLF4 chromatin complex was immunoprecipitated by the anti-KLF4 antibody and then the target protein was eluted. In the experiment, anti-IgG (Santa Cruz, CA, USA) served as a control.

### 2.16. Statistical Analysis

The data were expressed as mean ± standard deviation. SPSS 22.0 (IBM, Armonk, USA) was utilized to data evaluation. Discrepancies between the two groups were assessed by *t*-test while those among multiple groups by one-way analysis of variance and Tukey's test. With *P* < 0.05, statistical significance was registered.

## 3. Results

### 3.1. KLF4 and miR-155-5p Are Overexpressed in Acute Renal Allograft Injury

**Figure 1:**
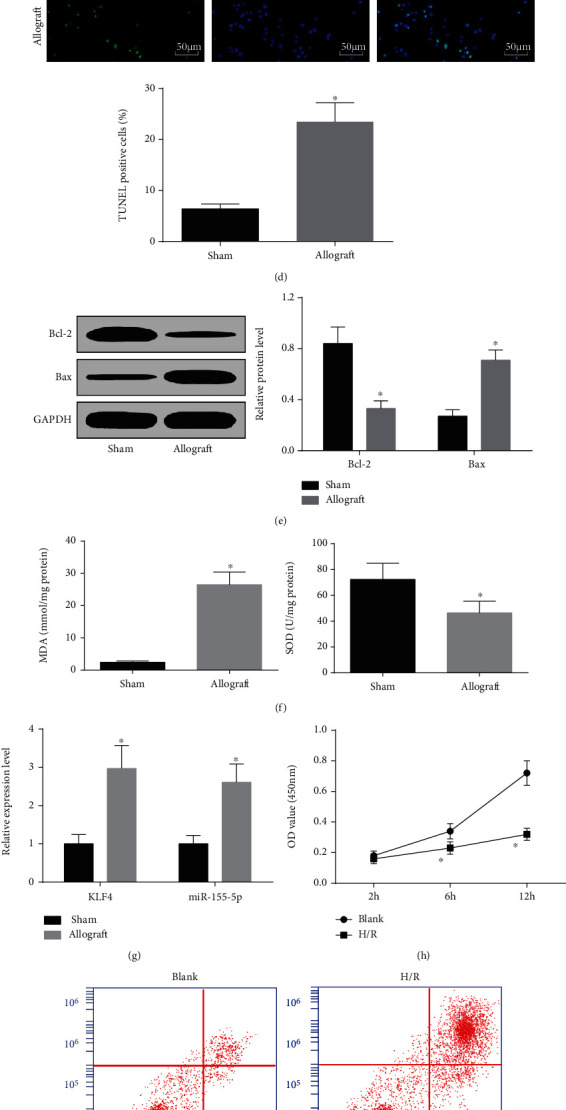
KLF4 and miR-155-5p are overexpressed in mice with acute renal allograft injury. (a) Serum creatinine and eGFR contents in mice. (b) ELISA detected serum IFN-*γ*, TNF-*α*, and IL-2 contents in mice. (c) H&E staining detected kidney injury of mice. (d) TUNEL staining detected renal tubular apoptosis of mice. (e) Western blot detected Bax and Bcl-2 protein expression in renal tissues of mice. (f) MDA content and SOD activity in renal tissues of mice. (g) RT-qPCR detected KLF4 and miR-155-5p expressions in renal tissues of mice. (h) CCK-8 assay detected proliferation of H/R-treated HK-2 cells. (i) Flow cytometry detected the apoptosis rate of H/R-treated HK-2 cells. (j) Western blot detected Bax and Bcl-2 protein expressions in H/R-treated HK-2 cells. (k) RT-qPCR detected KLF4 and miR-155-5p expressions in H/R-treated HK-2 cells. The data were expressed as mean ± standard deviation; ^∗^*P* < 0.05 compared with the sham/blank group. (a–g) *n* = 5 and (h–k) *n* = 3.

Renal function was evaluated by detecting serum creatinine and eGFR. In mice with acute renal allograft, serum creatinine level was increased while eGFR level was decreased. At the same time, IFN-*γ*, TNF-*α*, and IL-2 contents in serum were detected to increase in mice after acute renal allograft (Figures 1(a) and 1(b)).

Detected by H&E staining, it was observed that renal tubules were severely damaged, some cells were arranged disorderly, renal tubules were dilated, and vacuoles were formed in mice with renal allograft (Figure 1(c)). Revealed by TUNEL staining and Western blot, mice with renal allograft showed increased TUNEL-positive rate and Bax level and reduced Bcl-2 level in renal tissues (Figures 1(d) and 1(e)), indicating cell apoptosis in mice after acute renal allograft. Oxidative stress damage of the kidney was measured by detecting MDA content and SOD activity. It was displayed that MDA content was heightened and SOD activity was impaired in mice with renal allograft injury (Figure 1(f)).

KLF4 is upregulated in AKI and knocking out KLF4 attenuates renal dysfunction and interstitial fibers in I/R mice [7]. Also, miR-155-5p has been explored to upregulate in the injured kidneys [22]. In this study, KLF4 and miR-155-5p expressions were increased in renal tissues after acute renal allograft (Figure 1(g)).

For further validation of the effects of KLF4 and miR-155-5p on kidney injury after acute allograft, an H/R model of HK-2 cells *in vitro* was established [18]. CCK-8 assay, flow cytometry, and Western blot were utilized to examine cell proliferation, apoptosis, and apoptosis-related proteins. The results revealed that proliferation capacity was impaired, apoptosis rate and Bax level were increased, and Bcl-2 level was suppressed in H/R-treated HK-2 cells (Figures 1(h)–1(j)). Also, in H/R-treated HK-2 cells, KLF4 and miR-155-5p levels were both upregulated (Figure 1(k)). Shortly, KLF4 and miR-155-5p were related to acute renal allograft injury.

### 3.2. KLF4 Binds to miR-155-5p Promoter

**Figure 2:**
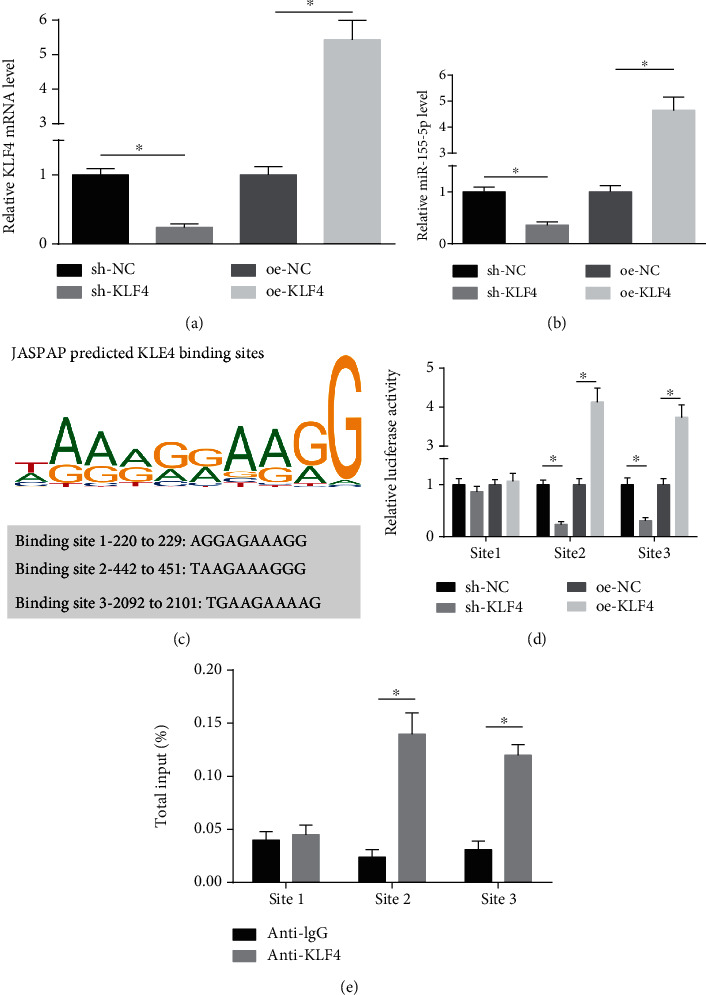
KLF4 binds to miR-155-5p promoter. (a and b) RT-qPCR detected KLF4 and miR-155-5p mRNA expressions in HK-2 cells. (c) JASPAR predicted the binding sites of KLF4 on miR-155-5p promoter. (d) Luciferase activity of oe-KLF4 and sh-KLF4 on miR-155-5p promoter reporters. (e) ChIP assay detected the site of KLF4 on miR-155-5p promoter. The data were expressed as mean ± standard deviation; ∗ represented *P* < 0.05; *N* = 3.

Though KLF4 and miR-155-5p expressions in acute renal allograft injury were determined, their internal action remained unclear. KLF4 is a transcription factor to bind to the promoter of miRNA [23]. Based on that, the same mechanism of KLF4 was speculated to work with miR-155-5p. To clarify the regulatory mechanism of KLF4 and miR-155-5p, we firstly interfered and overexpressed KLF4 in the cells (Figure 2(a)) and then observed that overexpressed/depleted KLF4 up-/downregulated miR-155-5p, proving that KLF4 positively regulated miR-155-5p (Figure 2(b)). Then, JASPAR database searched 3 potential binding sites of KLF4 on the promoter of miR-155-5p (Figure 2(c)). Subsequently, each KLF4 binding site was cloned into pGL3-Basic vector to analyze KLF4-regulated miR-155-5p promoter region. The findings suggested that KLF4 at sites 2 and 3 substantially regulated miR-155-5p while KLF4 at site 1 did not (Figure 2(d)). In addition, ChIP assay confirmed that KLF4 could directly bind to the miR-155-5p promoter and KLF4 was recruited through the binding sites 2 and 3 (Figure 2(e)).

### 3.3. Depleting miR-155-5p Attenuates Acute Renal Allograft Injury

**Figure 3:**
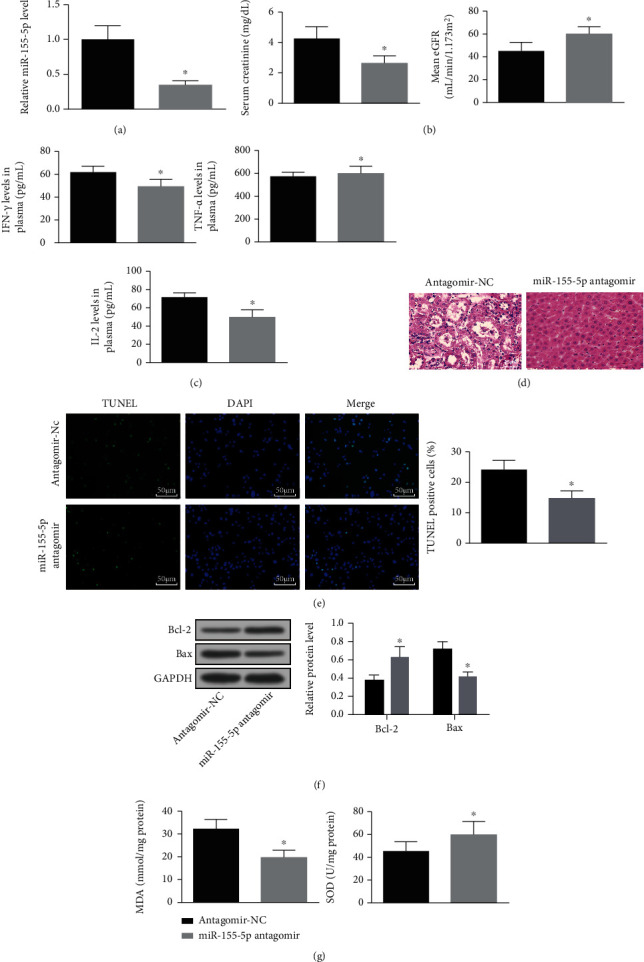
Depleting miR-155-5p attenuates acute renal allograft injury in mice. (a) RT-qPCR detected miR-155-5p expression in renal tissues of mice. (b) Serum creatinine and eGFR contents of mice. (c) ELISA detected serum IFN-*γ*, TNF-*α*, and IL-2 contents in mice. (d) H&E staining detected kidney injury of mice. (e) TUNEL staining detected renal tubular apoptosis of mice. (f) Western blot detected Bax and Bcl-2 protein expression in renal tissues of mice. (g) MDA content and SOD activity in renal tissues of mice. The data were expressed as mean ± standard deviation; ^∗^*P* < 0.05 compared with the antagomir-NC group, *n* = 5.

To explore the effects of miR-155-5p inhibition on acute renal allograft injury, miR-155-5p expression was interfered by miR-155-5p antagomir in mice with acute renal allograft (Figure 3(a)). Then, it was found that miR-155-5p antagomir treatment could reduce serum creatinine, IFN-*γ*, TNF-*α*, and IL-2 levels and elevated eGFR level in serum (Figures 3(b) and 3(c)). Moreover, downregulating miR-155-5p ameliorated renal tubular injury and reduced apoptosis and oxidative stress in renal tissues (Figures 3(d)–3(g)). It was implied that miR-155-5p knockdown attenuated acute renal allograft injury.

### 3.4. Downregulating miR-155-5p Facilitates H/R-Treated HK-2 Cell Proliferation and Suppresses Apoptosis

**Figure 4:**
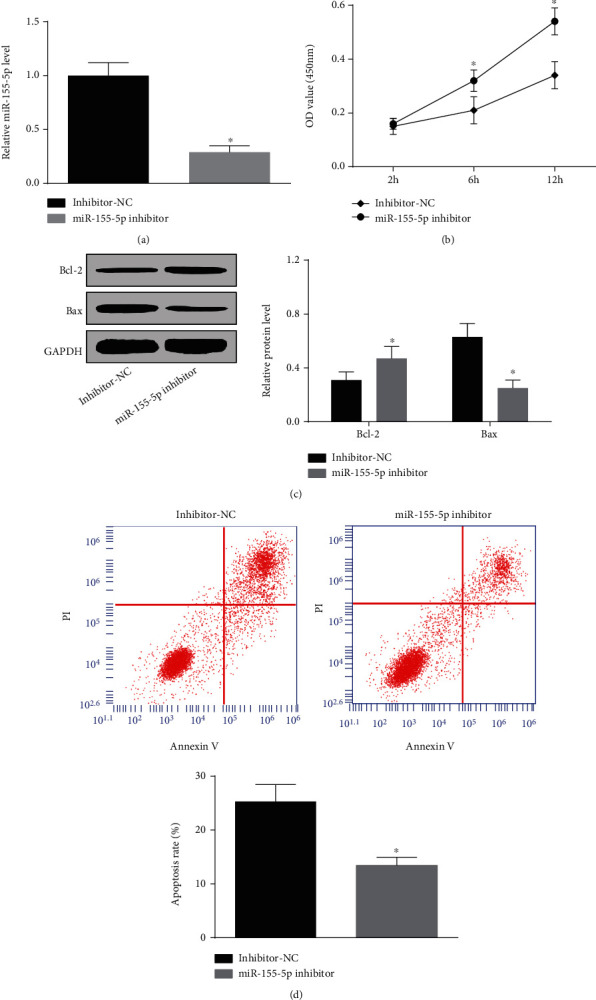
Downregulating miR-155-5p facilitates H/R-treated HK-2 cell proliferation and represses apoptosis. (a) RT-qPCR detected miR-155-5p expression in H/R-treated HK-2 cells. (b) CCK-8 assay detected the proliferation of H/R-treated HK-2 cells. (c) Flow cytometry detected the apoptosis rate of H/R-treated HK-2 cells. (d) Western blot detected Bax and Bcl-2 expressions in H/R-treated HK-2 cells. The data were expressed as mean ± standard deviation; ^∗^*P* < 0.05 compared with the inhibitor-NC group, *N* = 3.

The protective effects of miR-155-5p depletion on acute renal allograft injury were proved in animal models; then, its effects on H/R-treated HK-2 cells were deciphered. miR-155-5p inhibitor was transfected into the cells to successfully downregulate miR-155-5p (Figure 4(a)). Next, functional assays presented that in response to miR-155-5p inhibitor treatment, cell proliferation was reinforced, apoptosis rate and Bax level were reduced, and Bcl-2 level was heightened (Figures 4(b)–4(d)). It was found that miR-155-5p silencing relieved H/R-induced damage to HK-2 cells.

### 3.5. miR-155-5p Targets ERRFI1

**Figure 5:**
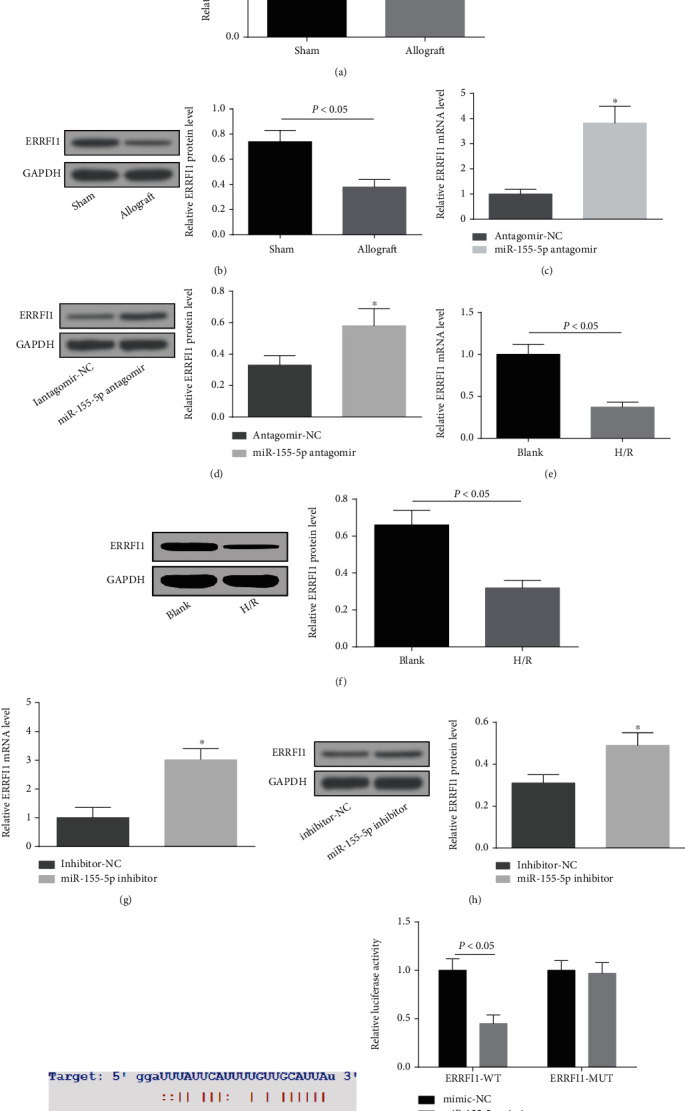
miR-155-5p targets ERRFI1. (a and b) RT-qPCR and Western blot detected ERRFI1 expression in renal tissues of mice after acute renal allografts. (c and d) RT-qPCR and Western blot detected ERRFI1 expression in renal tissues of mice after downregulation of miR-155-5p. (e and f) RT-qPCR and Western blot detected ERRFI1 expression in H/R-treated HK-2 cells. (g and h) RT-qPCR and Western blot detected ERRFI1 expression in H/R treated HK-2 cells after downregulation of miR-155-5p. (i) Starbase website predicted the binding site of miR-155-5p and ERRFI1. (j) Dual luciferase reporter gene assay verified the targeting relation between miR-155-5p and ERRFI1. The data were expressed as mean ± standard deviation; in mice, ^∗^*P* < 0.05 compared with the antagomir-NC group; In H/R-treated HK-2 cells and ^∗^*P* < 0.05 compared with the inhibitor-NC group. (a–d) *n* = 5 and (e–j) *n* = 3.

Detected by RT-qPCR and Western blot, ERRFI1 expression was downregulated in renal tissues of mice after acute renal allograft and in H/R-treated HK-2 cells, and moreover, downregulating miR-155-5p suppressed ERRFI1 level (Figures 5(a)–5(h)). Starbase website predicted the binding sites between miR-155-5p and ERRFI1 (Figure 5(i)). Dual luciferase reporter experiment made it clear that cotransfection of ERRFI1-WT and miR-155-5p mimic weakened the luciferase activity of HK-2 cells, while that of ERRFI1-MUT and miR-155-5p mimic did not (Figure 5(j)). All of those results hinted that ERRFI1 was targeted by ERRFI1.

### 3.6. Knocking Down ERRFI1 Antagonizes the Effects of Downregulated miR-155-5p on Acute Renal Allograft Injury

**Figure 6:**
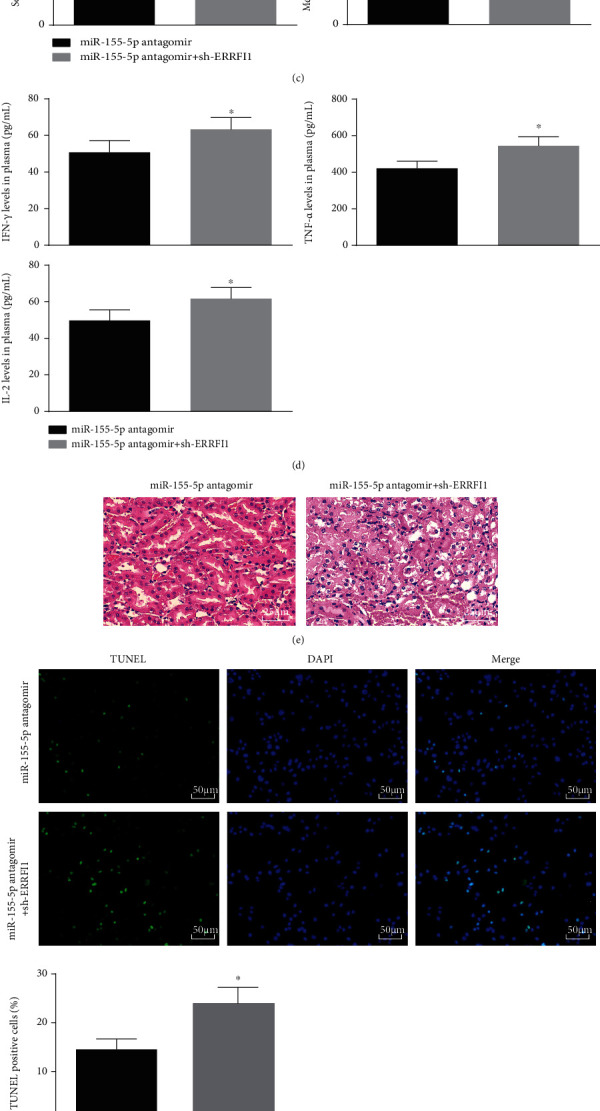
Knocking down ERRFI1 antagonizes the effects of downregulated miR-155-5p on acute renal allograft injury. (a and b) RT-qPCR and Western blot detected ERRFI1 expression in renal tissues of mice. (c) Serum creatinine and eGFR contents in mice. (d) ELISA detected serum IFN-*γ*, TNF-*α*, and IL-2 contents in mice. (e) H&E staining detected kidney injury of mice. (f) TUNEL staining detected renal tubular apoptosis of mice. (g) Western blot detected Bax and Bcl-2 protein expressions in renal tissues of mice. (h) MDA content and SOD activity in renal tissues of mice. The data were expressed as mean ± standard deviation; ^∗^*P* < 0.05 compared with the miR-155-5p antagomir group, *n* = 5.

The impacts of spontaneous knockdown of miR-155-5p and ERRFI1 on acute renal allograft injury were tested. It was uncovered that downregulating ERRFI1 impaired the effects of downregulated miR-155-5p on ERRFI1 expression. Besides, depleting ERRFI1 antagonized the impacts of miR-155-5p knockdown on serum creatinine, eGFR, IFN-*γ*, TNF-*α* and IL-2 levels, renal tubular injury, apoptosis, and oxidative stress (Figures 6(a)–6(h)).

### 3.7. Knocking Down ERRFI1 Offsets the Effects of Downregulated miR-155-5p on H/R-Treated HK-2 Cell Proliferation and Apoptosis

**Figure 7:**
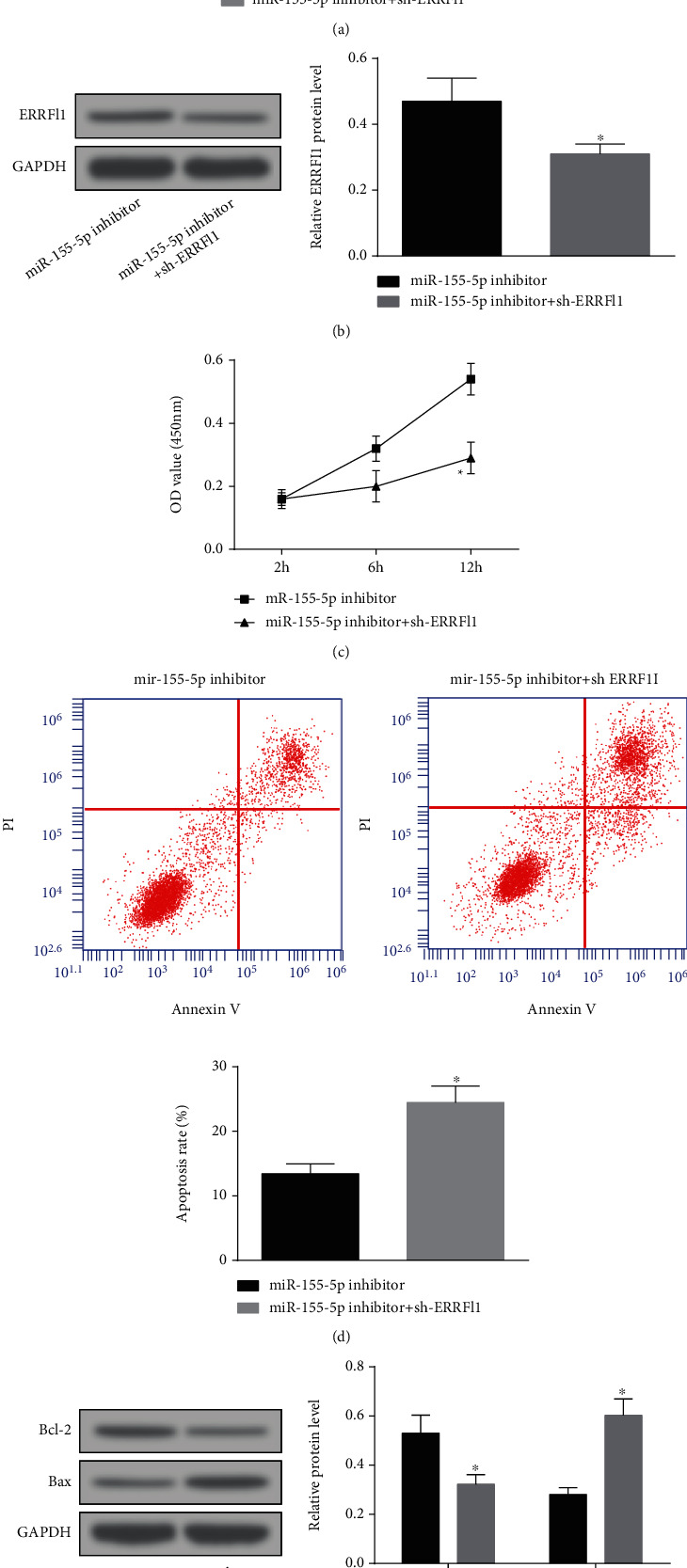
Knocking down ERRFI1 offsets the effects of downregulated miR-155-5p on H/R-treated HK-2 cell proliferation and apoptosis. (a and b) RT-qPCR and Western blot detected ERRFI1 expression in H/R-treated HK-2 cells. (c) CCK-8 assay detected the proliferation of H/R-treated HK-2 cells. (d) Flow cytometry detected the apoptosis rate of H/R-treated HK-2 cells. (e) Western blot detected Bax and Bcl-2 protein expression in H/R-treated HK-2 cells. The data were expressed as mean ± standard deviation; ∗ compared with the miR-155-5p inhibitor group, *N* = 3.

Lastly, the changes of HK-2 cell proliferation and apoptosis after depletion of miR-155-5p and ERRFI1 were observed. It was manifested that on the basis of miR-155-5p downregulation, further treatment of ERRFI1 depletion lowered ERRFI1 expression. Meanwhile, ERRFI1 knockdown rescued the effects of miR-155-5p downregulation on the proliferation and apoptosis of H/R-treated HK-2 cells (Figures 7(a)–7(f)).

## 4. Discussion

Kidney injury, specially IR/I-induced kidney injury, is the common result during acute renal allograft [24]. In this manuscript, we navigated and unveiled the mechanism of KLF4 in acute renal allograft injury. To begin with, we checked KLF4 and miR-155-5p expressions, and the findings presented that both the two were overexpressed in mouse renal allografts and H/R-treated HK-2 cells. Afterwards, we explored the interaction between KLF4 and miR-155-5p and found that KLF4 bound to the promoter of miR-155-5p. Subsequently, functional experiments discovered that depleting miR-155-5p reduced serum inflammation and attenuated oxidative stress, renal tubular injury, and apoptosis in mice with acute renal allograft injury, as well as facilitated proliferation and repressed apoptosis of H/R-treated HK-2 cells. After that, the downstream targets of miR-155-5p were predicted, and eventually ERRFI1, the downregulated gene in mice with acute renal allograft injury, was picked. Finally, knocking down ERRFI1 was detected to antagonize the effects of downregulated miR-155-5p on mice with acute renal allograft injury, as well as on H/R-treated HK-2 cell proliferation and apoptosis.

KLF4 level was examined to upregulate in mice with IR/I in the kidney, and knocking down KLF4 prevented kidney from further dysfunction, while overexpressing KLF4 oppositely worked [7]. Also, another work has elucidated that in response to IR/I, KLF4 level went to an upregulation in the kidney of mice [6]. KLF4 expression in astrocytes was induced within 3 d of ischemia, as well as in oxygen-glucose deprivation-treated rat primary cortical astrocytes [25]. As to the interaction between KLF4 and miR-155-5p, nearly no study has reported the binding of KLF4 on the promoter of miR-155-5p, which requires further validation.

Impressively, raised miR-155-5p expression in urine suggested the rejection after renal transplantation [26]. Also, miR-155-5p was overexpressed in oxalate-/calcium-induced oxidative stress injury in the kidney, and downregulating miR-155-5p attenuated oxidative stress, as suggested by reduced MDA content [27]. In a similar way, miR-155 inhibition could partially encourage viability and hinder inflammation, oxidative stress, and apoptosis of HK-2 cells in sepsis-induced AKI [28]. In compliance with the discoveries, in LPS-induced AKI, elevated miR-155 expression was recognizable in tubular epithelial cells, and miR-155 suppression partially attenuated inflammation and apoptosis, which was reflected by decreased TNF-*α* content [13]. Experimentally, miR-155 expression suggested an increase in rats with I/R-induced AKI and H/R-treated cells, and moreover, restored miR-155 enhanced apoptosis and discouraged proliferation of H/R-treated NRK-52E cells, while depleted miR-155 had the opposite functions [14]. In the context of abnormal allograft, miR-155 level in plasma was elevated, which was connected with acute rejection of renal allografts in rats [11]. Besides, the upregulated miR-155 accelerated LPS-induced tubular cell apoptosis, while suppressing miR-155 at least contributed to attenuated AKI [29]. Further echoed without research, another literature has elaborated that miR-155 expression reached a high level in renal tissues and HK-2 cells after chronic intermittent hypoxia treatment, and elevating miR-155 augmented oxidative stress in renal tubular cells [30]. In renal I/RI, miR-155 expression went toward an increase in rat renal tissues and HR-treated HK-2 cells, and restoring miR-155 strengthened inflammation response in HR-treated HK-2 cells [20]. At present, no reported research has declared the relation between miR-155-5p and ERRFI1. In fact, ERRFI1 level was insufficiently expressed in AKI, and ERRFI1 elevation mediated by miR-152-3p was inhibitory for the apoptotic and inflammatory activities of renal cells [15]. However, the more specific mechanism of ERRFI1 in kidney injury after acute renal allograft needs more researches for comprehensive explanation.

All in all, it was elucidated that silencing KLF4 mediated miR-155-5p to enhance ERRFI1 expression, thereby attenuating acute renal allograft injury in mice, as well as promoting proliferation and suppressing apoptosis of H/R-treated HK-2 cells. This work more or less widened our horizon to the mechanism of KLF4/miR-155-5p/ERRFI1 axis in acute renal allograft injury, which supplied a novel approach to manage acute renal allograft injury. However, whether the KLF4/miR-155-5p/ERRFI1 axis works in other diseases needs more explorations.

## Figures and Tables

**Table 1:** Primer sequences.

Genes	Primer sequences
KLF4	5′-GCGGGAAGGGAGAAGACAC-3′
	5′-GGGGAAGACGAGGATGAAGC-3′
miR-155-5p	5′-GCTTCGGTTAATGCTAATCGTG-3′
	5′-CAGAGCAGGGTCCGAGGTA-3′
ERRFI1	5′-CACGGCGCAGCCTCACTCTG-3′
	5′-CTTGGGGCAGGGGCTCTTGAC-3′
GAPDH	5′-TCCCATCACCATCTTCCA-3′
	5′-CATCACGCCACAGTTTTCC-3′
U6	5′-GGAACGATACAGAGAAGATTAGC-3′
	5′-TGGAACGCTTCACGAATTTGCG-3′

Note: KLF4: Kruppel-like factor 4; miR-155-5p: microRNA-155-5p; ERRFI1: ERBB receptor feedback inhibitor 1; GAPDH: glyceraldehyde-3-phosphate dehydrogenase.

## Data Availability

The analyzed data sets generated during the study are available from the corresponding author on reasonable request.
